# Not just another genome

**DOI:** 10.1186/1741-7007-9-8

**Published:** 2011-02-04

**Authors:** Diethard Tautz

**Affiliations:** 1 MPI for Evolutionary Biology, Plön, Germany

## Abstract

Sequence analysis of the *Daphnia pulex *genome holds some surprises that could not have been anticipated from what was learned so far from other arthropod genomes. It establishes *Daphnia *as an eco-genetical model organism *par excellence*.

## Genome of an aquatic sensor

**Figure 1:**
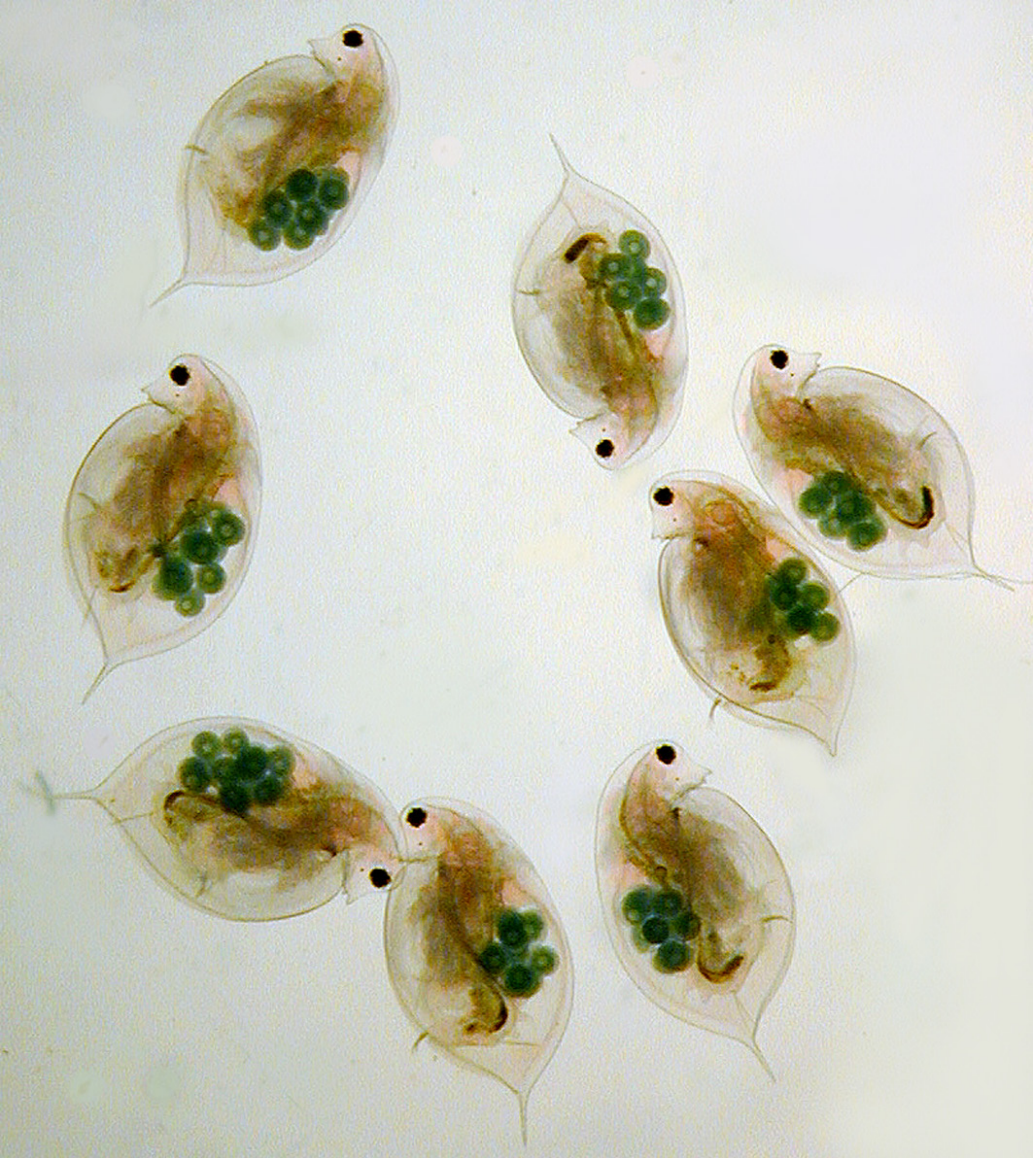
**Parthenogenetic *Daphnia***. Individuals of *Daphnia pulex *derived from a parthenogenetic line kept in a flow-through system that generates constant environmental conditions. The animals are highly synchronized, both with respect to size and the number of eggs they produce. This demonstrates the excellent experimental accessibility of *Daphnia *as a fresh water ecological model system (Reproduced by kind permission of Winfried Lampert, taken from '*Daphnia*: Development of a Model Organism in Ecology and Evolution' [13]).

One of my first courses in biology included an experiment in which we subjected *Daphnia *populations in glass beakers to different concentrations of a water pollutant and counted the fraction that stopped swimming around. This is still a very common test to monitor water quality and it is said to be more sensitive than any conventional chemical analysis. It reflects the fact that *Daphnia *plays a key role in fresh water ecology (Figure 1), not only for testing but also as a central component of nutrient cycles. In a typical lake in spring, *Daphnia *lives in paradise with food in the form of algae swimming around it ready to be collected at will. But no paradise lasts for ever. As the *Daphnia *population grows, algae become increasingly rare to the point of almost complete disappearance, leading to a short phase in the yearly cycle of a lake in which the water becomes crystal clear [1]. *Daphnia *itself increasingly becomes a victim of predators during the yearly cycle and, although it starts to develop defenses [2], its population shrinks such that the algae can become more abundant again. The nutrient flux that is involved in this cycle is enormous and drives the whole ecology of the lake. But how can a genome sequence help to learn more about this ecology? The *Daphnia *genome [3] turns out to hold a large number of genes which were previously not known and which, excitingly, are likely to have a specific role in the interaction with its environment. The *Daphnia *genome may thus become a Rosetta stone for studying the genetic repertoire of fresh water ecology.

## An abundance of newly evolved genes

Could one have expected that specific genes have evolved to cope with particular environmental challenges? A common notion in current evolutionary biology is that novelty arises out of the duplication and redeployment of ancient genes or protein domains in new regulatory pathways. This would not leave much room for the evolution of completely new genes. However, it is well known that every major evolutionary lineage contains genes that are only found in this lineage and can therefore be expected to have newly evolved [4]. There are also good reasons to speculate that they have lineage specific functions [4] that likely include some that are relevant for environmental interactions. And it is now clear that *Daphnia *has a particularly large number of newly evolved genes - roughly a third of all that were identified in its genome so far.

One focus of the genome paper [1] is therefore to trace the origin of this novel gene repertoire. A hallmark of these genes is that they occur in gene families - that is, they have arisen, duplicated and diverged within the genome. The authors find a generally higher propensity for gene duplications and retention in *Daphnia *compared to other organisms and this includes not only the unknown genes, but also well known ones. For example, the expansion of photoresponsive gene families such as cryptochromes, opsins, and G proteins may play a role in coping with the complex light regime in aquatic environments [1]; or multiple copies of insulin receptor genes with special structures and differential expression might contribute to the plasticity in body size, as suggested in a companion paper [5]. Of course, gene duplication on its own does not create much novelty, but these duplicates diverge over time, with respect to both their regulation and their sequences. And there is abundant evidence for concerted evolution among duplicates [1], which may create an additional level of evolutionary dynamics [6]. Interestingly, in *Daphnia *there is also a large fraction of very recent duplicates that show differential expression to environmental challenges, indicating that these duplicates have very quickly become involved in specific adaptations [1]. Again, one has to emphasize that this includes homologues of both previously known and unknown genes; thus, regulatory and protein evolution appear to take place concurrently. In fact, this tendency was already found in a previous study that aimed to identify cadmium-responsive genes [7]. Among the genes that were differentially regulated in response to varying doses of cadmium were metallothionein genes with highly diverged protein sequences, but with consensus metal-responsive elements in their promotors.

A clear case for an adaptive role of duplication and selective retention of duplicates comes from the analysis of genes involved in known metabolic pathways. Half of the expanded metabolic genes were found to belong to a subset of seven distinct pathways and the expression patterns of these genes co-diverge according to their pathway, not to their evolutionary history [1]. But also the non-classifiable genes show expression characteristics that suggest a direct connection to the environment. When differential expression with respect to different environmental conditions, such as exposure to metals or to predators, was analyzed, twice as many of them were responsive compared to the set of known genes [1]. Most intriguingly, there is apparently a further set of transcripts that are responsive to the environment, but whose structure could not yet be described since they were not found in EST libraries and did not fit the gene annotation models. They were identified by hybridization of RNA to whole genome tiling arrays, which revealed almost 35,000 additional transcriptionally active regions in the genome. These were not captured by other identification methods, but likely represent exons of an unknown number of additional genes [1] that await structural and functional characterization.

## Life cycle plasticity may facilitate rapid evolution

Why is *Daphnia *so particularly amenable to developing an eco-responsive gene repertoire? On the one hand it is known that positive selection is much more efficient in large populations, since even genes or alleles that provide only a very small advantage are retained in the population rather than being lost by drift. However, large population sizes are also found in the insects for which full genome sequences are available - and they show no indication of a particularly strong genomic response to the environment. The explanation may lie with some additional peculiarities of population genetics shown by *Daphnia*. It can switch between parthenogenetic and sexual reproduction and it can produce resting stages that can survive for decades [2]. Since parthenogenetic reproduction is numerically twice as efficient as sexual reproduction, *Daphnia *takes advantage of this in spring when a particularly fast population expansion is possible. This leads to a rapid amplification of clones that may have only a minimal advantage over their conspecifics. Of course, once the environment changes, the advantage of such clones may falter quickly, but this is the point where they can go into a sexual cycle and can produce special eggs that are protected by a cuticular structure that allows them to survive in the mud [2]. Thus, all lakes harbor a genetic reservoir of resting eggs derived from animals that had a particular advantage at a previous time. Genes or alleles that were once successful can thus be preserved, even if the environmental conditions are temporarily changed. An explicit evolutionary theory that models the long-term adaptive consequences of such complex life cycles is still missing but, at least intuitively, it would seem that this adds to the evolutionary dynamics that have led to the special gene repertoire of *Daphnia*.

Because of these peculiarities, *Daphnia *should now also become a prime model for studying the evolution and the role of sex. One of the companion papers has indeed already specifically addressed such issues by looking at the evolutionary dynamics of transposons in *Daphnia *[8]. These authors identified the major transposon families in the *Daphnia *genome and found active copies for most of them. Six of these were then studied in lines where sex was either promoted or inhibited. The data indicate that sexual reproduction is indeed a major factor to keep the elements under control. This effect could at least partially compensate for the short-term cost of sex and thus explain why sexual reproduction is maintained [8]. Intriguingly, a previous study had suggested that sexual and parthenogenetic reproduction makes use of the same set of meiosis related genes and that an expansion of this gene complement may have helped to develop the parthenogenetic life cycle [9].

Thus, both the ecological relevance and the evolutionary dynamics of *Daphnia *populations are bound to attract general attention to *Daphnia *as a new model system in genetics. Duly, it is already listed among the small select group of model organisms for biomedical research at the NIH [10]. The current genome paper focuses on *D. pulex *but another species of the genus, *Daphnia magna*, has an equally long history in ecological research and efforts to elucidate its genome are underway as well [11]. These developments are bound to fuel the newly emerging discipline of ecological genomics [12], which has so far been one of the last black boxes of genetic research.
